# Supplementary material for: Characterization of neutralizing antibody with prophylactic and therapeutic efficacy against SARS-CoV-2 in rhesus monkeys
Source: Nat Commun. 2020 Nov 13;11:5752. doi: 10.1038/s41467-020-19568-1 (PMC7666115; doi:10.1038/s41467-020-19568-1)
Supplement: Supplementary file 1 — Supplementary Information [file 41467_2020_19568_MOESM1_ESM.pdf]

Supplementary Information

Characterization of neutralizing antibody with prophylactic and therapeutic efficacy against SARS-CoV-2 in rhesus monkeys

Wang et al.

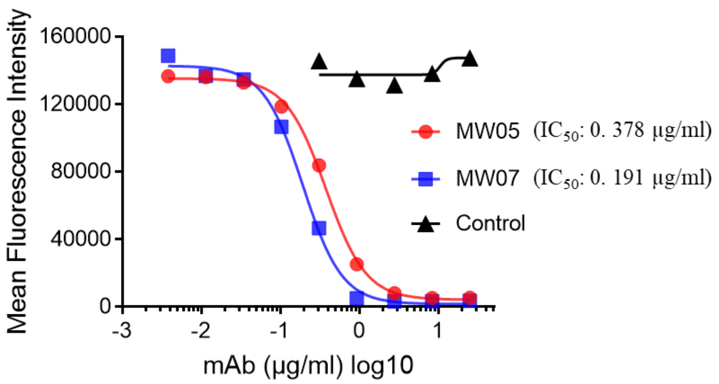

Supplementary Fig. 1 The abilities of MW05 and MW07 to block SARS-CoV-2 RBD interaction with ACE2 overexpressing HEK293 cells was evaluated by FACS. Pre-incubated mAb and SARS-CoV-2 RBD-mFc mixtures were added to ACE2 overexpressing HEK293 cells to check the binding of RBD to ACE2. Goat anti mouse IgG Fc-FITC was used as secondary antibody. Irrelevant human IgG1 was used as control.

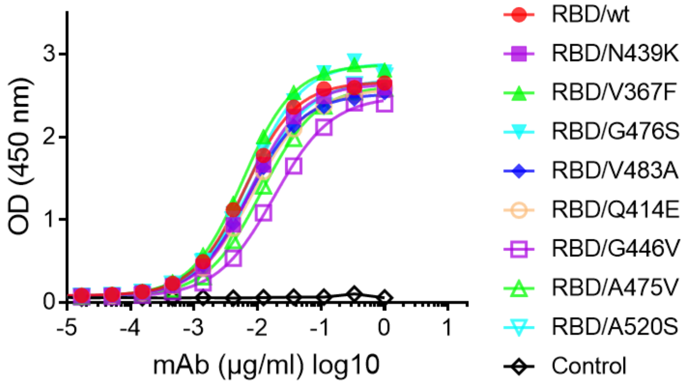

Supplementary Fig. 2 Binding of ACE2 to different SARS-CoV-2 RBD mutants by ELISA. RBD recombinant proteins were coated on 96-well plates. Human ACE2-mFc was then added into plates to check the binding.

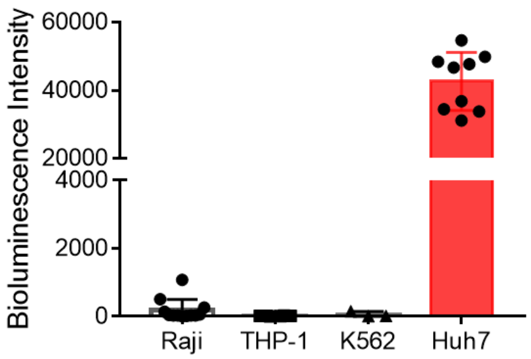

Supplementary Fig. 3 The infection of SARS-CoV-2 pseudovirus in Raji, THP-1, K562 and Huh7 cells. The average ± SD from n=10 of three independent experiments with technical duplicates is shown.

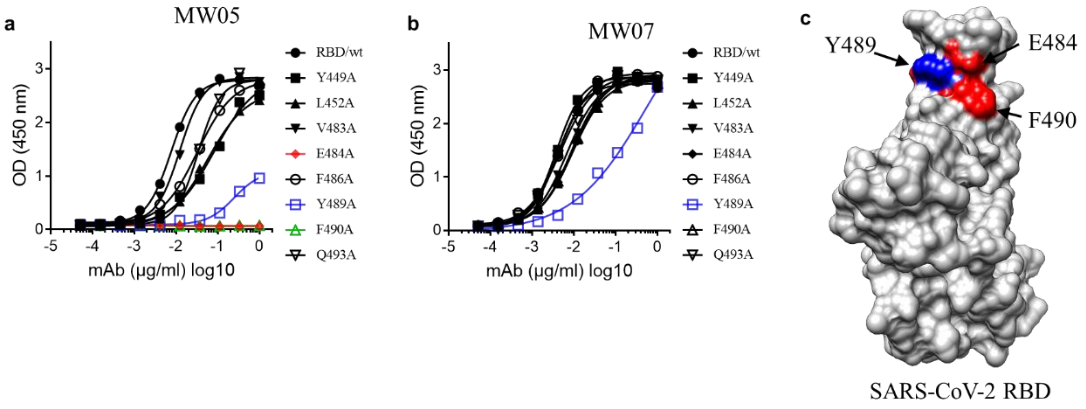

Supplementary Fig. 4 Binding of MW05 and MW07 to SARS-CoV-2 RBD. a Binding of MW05 to mutants was performed by ELISA. **b** Binding of MW07 to mutants was performed by ELISA. **c** Highlighting the key amino acid residues on SARS-CoV-2 RBD structure.

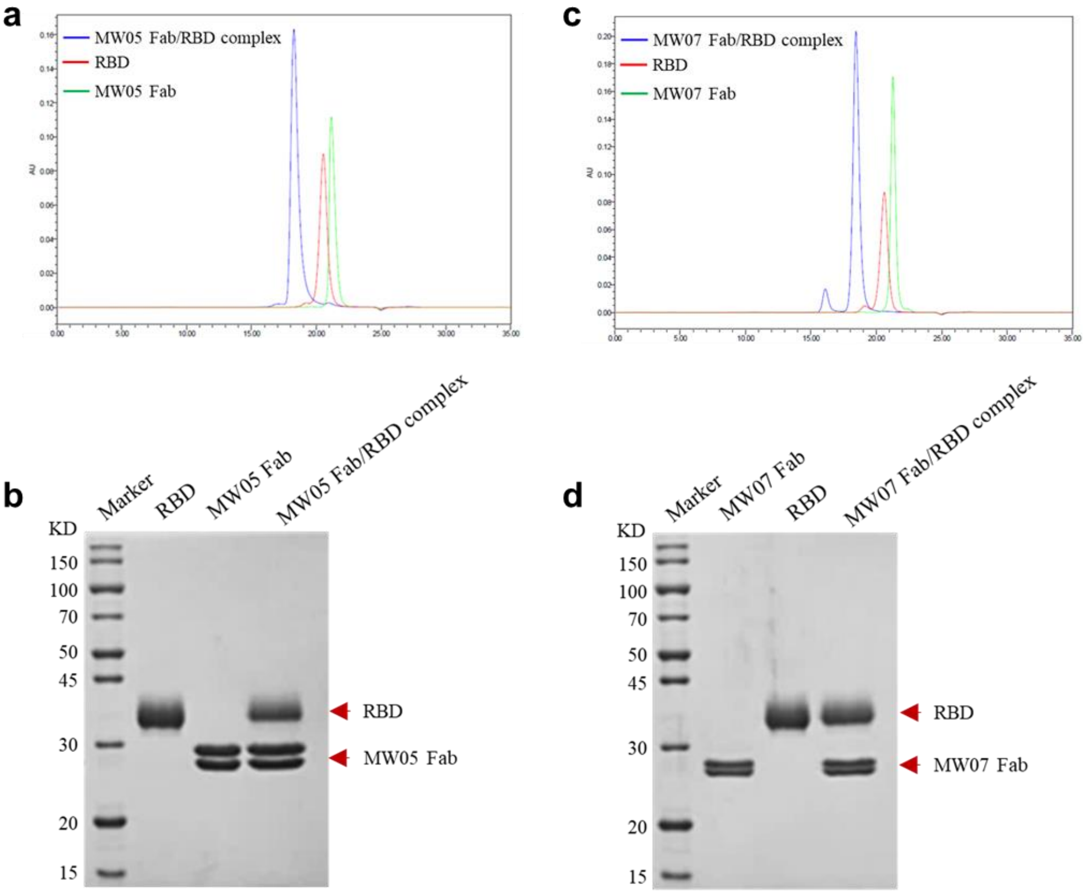

Supplementary Fig. 5 Generation and identification of SARS-CoV-2 RBD/MW05 Fab and SARS-CoV-2 RBD/MW07 Fab complexes. a Characterization of RBD, MW05 Fab and RBD/MW05 Fab complex by HPLC. **b** Characterization of RBD, MW05 Fab and RBD/MW05 Fab complex by SDS-PAGE. **c** Characterization of RBD, MW07 Fab and RBD/MW07 Fab complex by HPLC. **d** Characterization of RBD, MW07 Fab and RBD/MW07 Fab complex by SDS-PAGE.

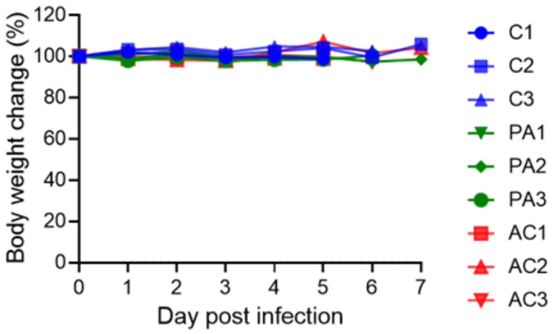

Supplementary Fig. 6 The body weight of each monkey was checked every day. C indicates the control group, PA indicates the pre-challenge group and AC indicates the post-challenge group.

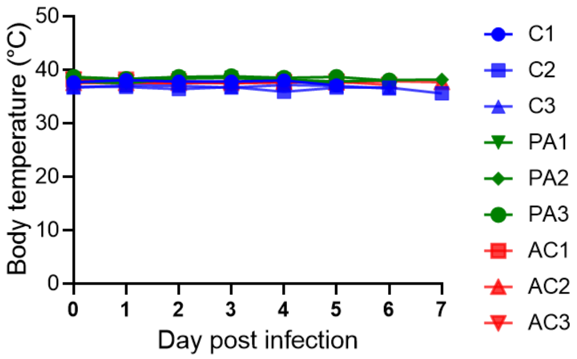

Supplementary Fig. 7 The body temperature of each monkey was checked every day. C indicates the control group, PA indicates the pre-challenge group and AC indicates the post-challenge group.

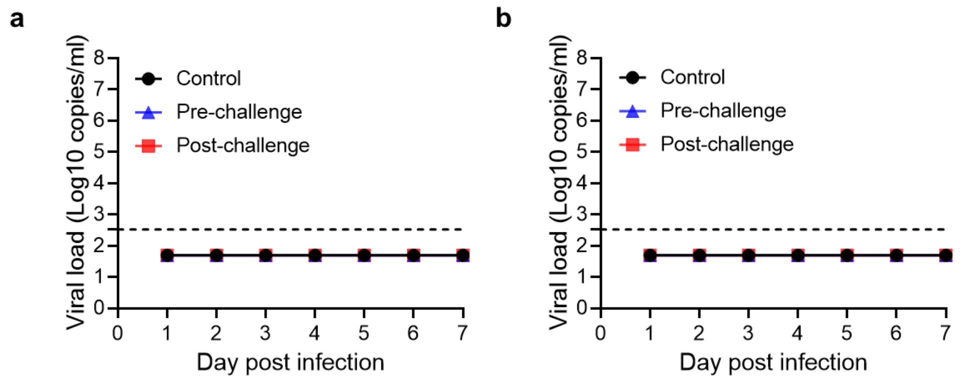

Supplementary Fig. 8 Viral titer of nasal swabs (a) or blood samples (b) of all monkeys were evaluated by qRT-PCR.

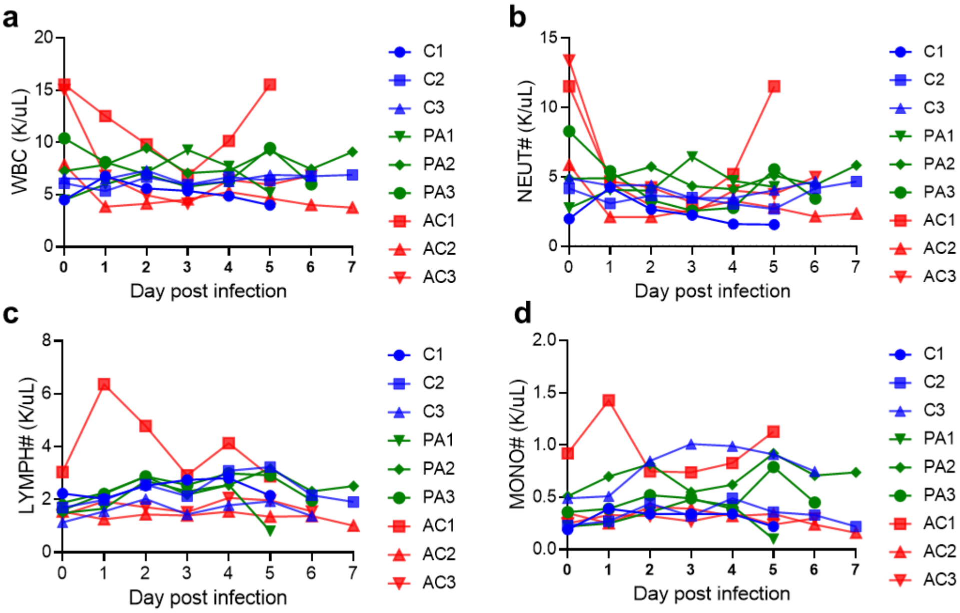

Supplementary Fig. 9 White blood cells (a), neutrophils (b), lymphocytes (c) and monocytes (d) in each monkey were monitored every day. C indicates the control group, PA indicates the pre-challenge group and AC indicates the post-challenge group.

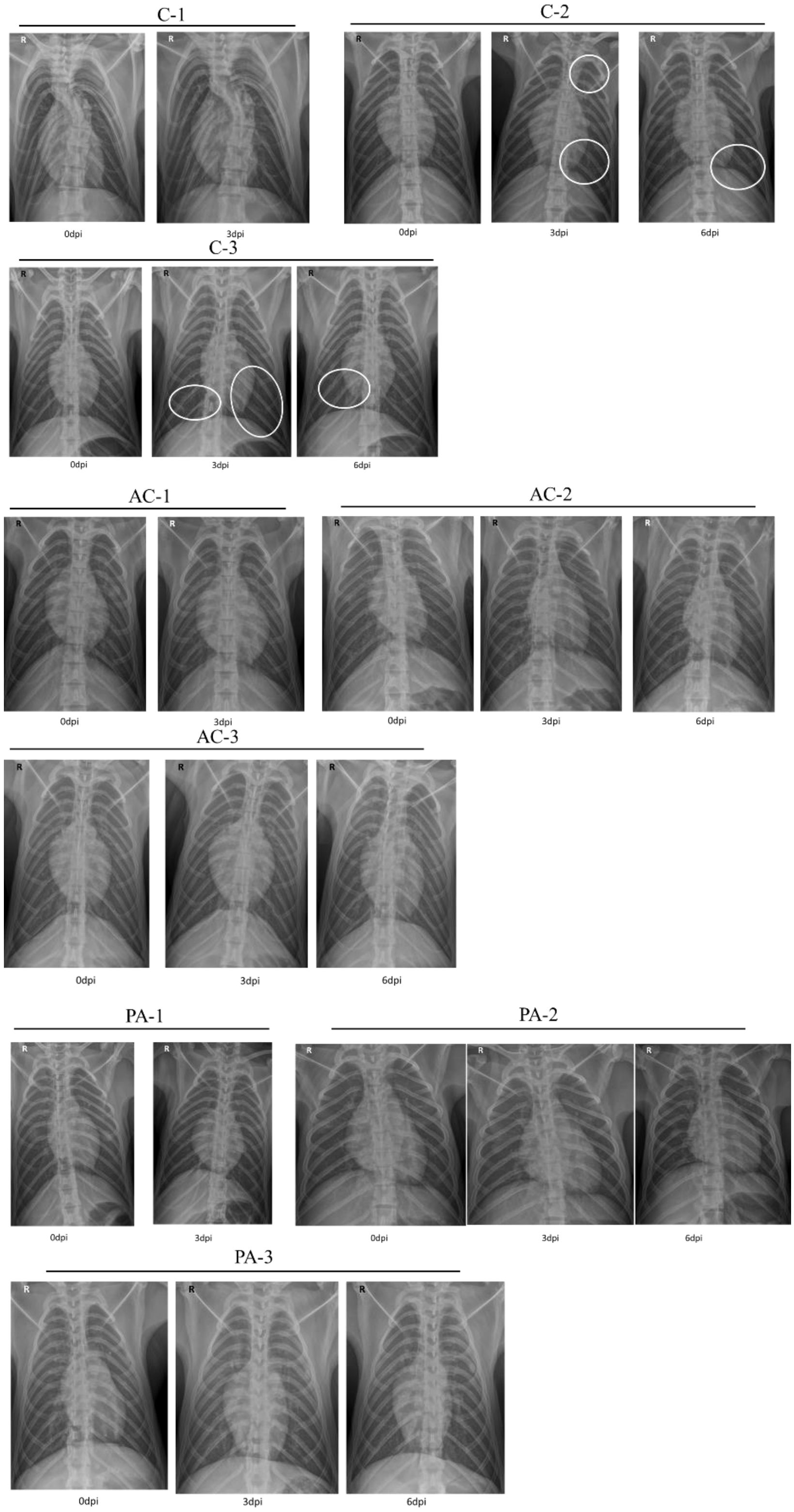

Supplementary Fig. 10 X-ray imaging of all animals in this study. C indicates the control group, PA indicates the pre-challenge (prophylactic) group and AC indicates the post-challenge (therapeutic) group.

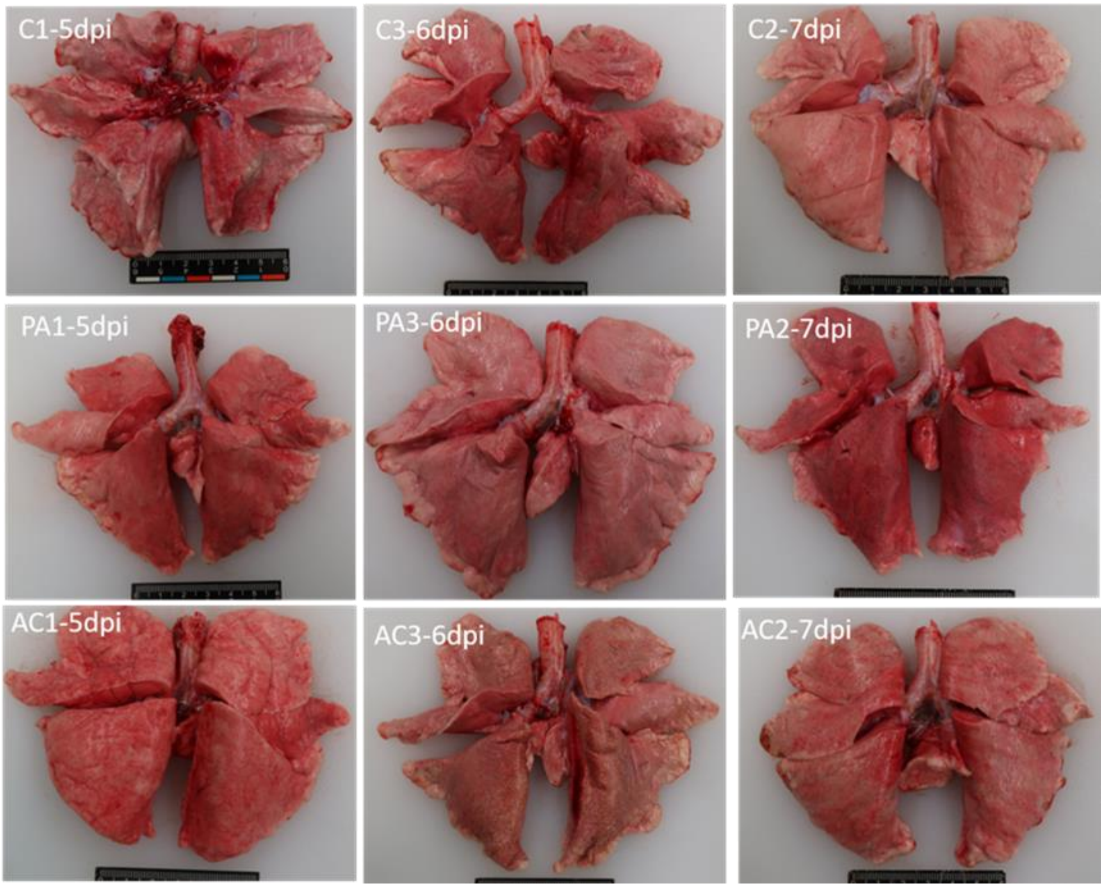

Extended Data Fig. 11 Whole Lung Photograph of Experimental Animals.

Supplementary Table. 1 Summary of histopathology analysis of animals in control, prophylactic and therapeutic Groups

Experimental group	Animal #	5 dpi						6 dpi						7 dpi					
		Inflammatory cells in alveolar wall	Alveolar wall thickening	Inflammatory exudate in alveolar cavity	Alveolar cavity fibrosis	Peri-bronchiolar inflammatory cells	Perivascular (small vessels) inflammatory cells	Inflammatory cells in alveolar wall	Alveolar wall thickening	Inflammatory exudate in alveolar cavity	Alveolar cavity fibrosis	Peri-bronchiolar inflammatory cells	Perivascular (small vessels) inflammatory cells	Inflammatory cells in alveolar wall	Alveolar wall thickening	Inflammatory exudate in alveolar cavity	Alveolar cavity fibrosis	Peri-bronchiolar inflammatory cells	Perivascular (small vessels) inflammatory cells
Control group	C1	++	++	++	+++	+	—	/	/	/	/	/	/	/	/	/	/	/	/
	C2	/	/	/	/	/	/	/	/	/	/	/	/	+	++	+	+	+	+
	C3	/	/	/	/	/	/	++	+++	+	+	+	+	/	/	/	/	/	/
	Overall grading	**++/+++ (moderate-severe)**						**++/+++ (moderate-severe)**						**++ (moderate)**					
Therapeutic group	AC1	++	++	+	++	+	—	/	/	/	/	/	/	/	/	/	/	/	/
	AC2	/	/	/	/	/	/	/	/	/	/	/	/	+++	+++	++	++	+	+
	AC3	/	/	/	/	/	/	++	++	++	++	+	+	/	/	/	/	/	/
	Overall grading	**+/++ (mild-moderate)**						**+/++ (mild-moderate)**						**++/+++ moderate-severe**					
Prophylactic group	PA1	++	++	+	+	+	+	/	/	/	/	/	/	/	/	/	/	/	/
	PA2	/	/	/	/	/	/	/		/	/	/	/	++	++	+	+	+	+
	PA3	/	/	/	/	/	/	+	+	+	+	+	—	/	/	/	/	/	/
	Overall grading	**+/++ (mild-moderate)**						**+ /- (no lesions/mild)**						**+/++ (mild-moderate)**					

Supplementary Table. 2 qPCR primers targeting SARS-CoV-2 S gene

Primer name	Primer sequence
SARS-CoV-2 RBD-qF1	5'-CAATGGTTTAACAGGCACAGG-3'
SARS-CoV-2 RBD-qR1	5'-CTCAAGTGTCTGTGGATCACG-3'
