# Supplementary material for: Characterization of neutralizing antibody with prophylactic and therapeutic efficacy against SARS-CoV-2 in rhesus monkeys
Source: Nat Commun. 2020 Nov 13;11:5752. doi: 10.1038/s41467-020-19568-1 (PMC7666115; doi:10.1038/s41467-020-19568-1)
Supplement: Supplementary file 3 — Reporting Summary [file 41467_2020_19568_MOESM3_ESM.pdf]

nature research

Corresponding author(s): Xun Gui

Last updated by author(s): Oct 14, 2020

Reporting Summary

Nature Research wishes to improve the reproducibility of the work that we publish. This form provides structure for consistency and transparency in reporting. For further information on Nature Research policies, see our Editorial Policies and the Editorial Policy Checklist.

Statistics

For all statistical analyses, confirm that the following items are present in the figure legend, table legend, main text, or Methods section.

n/a	Confirmed	
☐	☒	The exact sample size (*n*) for each experimental group/condition, given as a discrete number and unit of measurement
☐	☒	A statement on whether measurements were taken from distinct samples or whether the same sample was measured repeatedly
☒	☐	The statistical test(s) used AND whether they are one- or two-sided<br>*Only common tests should be described solely by name; describe more complex techniques in the Methods section.*
☒	☐	A description of all covariates tested
☒	☐	A description of any assumptions or corrections, such as tests of normality and adjustment for multiple comparisons
☐	☒	A full description of the statistical parameters including central tendency (e.g. means) or other basic estimates (e.g. regression coefficient) AND variation (e.g. standard deviation) or associated estimates of uncertainty (e.g. confidence intervals)
☐	☒	For null hypothesis testing, the test statistic (e.g. F, t, r) with confidence intervals, effect sizes, degrees of freedom and P value noted<br>*Give P values as exact values whenever suitable.*
☒	☐	For Bayesian analysis, information on the choice of priors and Markov chain Monte Carlo settings
☒	☐	For hierarchical and complex designs, identification of the appropriate level for tests and full reporting of outcomes
☒	☐	Estimates of effect sizes (e.g. Cohen's *d*, Pearson's *r*), indicating how they were calculated

Our web collection on statistics for biologists contains articles on many of the points above.

Software and code

Policy information about availability of computer code

Data collection	Biacore S200 Control Software Version: 1.1, MOLECULAR DEVICES SoftMax Pro 7.0, CytExpert Software Version 2.4.0.28, Applied Biosystems StepOne Software Version 2.3. Ascent Software for Multiskan Version 2.6.
Data analysis	GraphPad Prism 7.00, CytoExpert software Version 2.4.0.28, BIAcore S200 Evaluation Software Version 1.1.

For manuscripts utilizing custom algorithms or software that are central to the research but not yet described in published literature, software must be made available to editors and reviewers. We strongly encourage code deposition in a community repository (e.g. GitHub). See the Nature Research guidelines for submitting code & software for further information.

Data

Policy information about availability of data

All manuscripts must include a data availability statement. This statement should provide the following information, where applicable:

- Accession codes, unique identifiers, or web links for publicly available datasets
- A list of figures that have associated raw data
- A description of any restrictions on data availability

All data have been included in the manuscript. Further information and requests for resources and reagents should be directed to and will be fulfilled by the corresponding author Xun Gui.

Field-specific reporting

Please select the one below that is the best fit for your research. If you are not sure, read the appropriate sections before making your selection.

☒ Life sciences ☐ Behavioural & social sciences ☐ Ecological, evolutionary & environmental sciences

For a reference copy of the document with all sections, see nature.com/documents/nr-reporting-summary-flat.pdf

Life sciences study design

All studies must disclose on these points even when the disclosure is negative.

Sample size	No statistical methods were used to predetermine sample size. For the animal study, nine rhesus monkeys divided to three groups (three in control group, three in prophylactic group and three in therapeutic group) were used for this study. The numbers of monkeys in each group meet the requirement for statistical analysis (at least 3 for each group), which is sufficient given the technical reproducibility.
Data exclusions	No data were excluded.
Replication	For the binding, neutralization and antibody-dependent enhancement assays, all experiments are reproducible. The Monkey experiments were not repeated in BSL-4 Lab.
Randomization	We divided nine monkeys (3 females and 6 males) into three groups, including the control group, the prophylactic group and the therapeutic group. Monkeys of the same sex were randomly divided into three groups.
Blinding	Animal studies were blinded to group allocation during data collection and analysis. For other experiments, data collection and analysis were performed by different people, the sample classification were replaced by simple marks during data analysis.

Reporting for specific materials, systems and methods

We require information from authors about some types of materials, experimental systems and methods used in many studies. Here, indicate whether each material, system or method listed is relevant to your study. If you are not sure if a list item applies to your research, read the appropriate section before selecting a response.

Materials & experimental systems

n/a	Involved in the study	
☐	☒	Antibodies
☐	☒	Eukaryotic cell lines
☒	☐	Palaeontology and archaeology
☐	☒	Animals and other organisms
☐	☒	Human research participants
☒	☐	Clinical data
☒	☐	Dual use research of concern

Methods

n/a	Involved in the study	
☒	☐	ChIP-seq
☐	☒	Flow cytometry
☒	☐	MRI-based neuroimaging

Antibodies

Antibodies used	Anti-CD16a /FITC, Sino Biological, Cat No: 10389-MM41-F, 10 μl/Test.<br>Anti-CD32a/FITC, Sino Biological, Cat No: 10374-MM02-F,10 μl/Test.<br>Anti-CD32b/c, Biolegend, Cat No: 398302, Clone No: S18005H.<br>Anti-CD64/FITC, Sino Biological, Cat No:10256-R401-F, 10 μl/Test.<br>Goat Anti-Human IgG Fc-HRP, Jackson ImmunoResearch, Cat No: 109-035-098, dilution: 1:5000.<br>Goat Anti-Mouse IgG Fc-HRP, Jackson ImmunoResearch, Cat No: 115-035-071, dilution: 1:5000.
Validation	We follow the manufacturer's instruction to use the above listed antibodies. All antibodies work well.<br>Anti-CD16a /FITC (Sino Biological, mouse, Specific to human CD16a, applicable for Flow Cytometry)<br>https://www.sinobiological.com/antibodies/cd16a-10389-mm41-f<br><br>Anti-CD32a/FITC (Sino Biological, mouse, Specific to human CD32a, applicable for Flow Cytometry)<br>https://www.sinobiological.com/antibodies/human-cd32a-10374-mm02-f<br><br>Anti-CD32b/c (Biolegend, mouse, Specific to human CD32b/c, applicable for Flow Cytometry)<br>https://www.biolegend.com/en-us/products/purified-anti-human-cd32bc-antibody-18270<br><br>Anti-CD64a/FITC (Sino Biological, rabbit, Specific to human CD64, applicable for Flow Cytometry)

https://www.sinobiological.com/antibodies/human-cd64-10256-r401-f

Goat Anti-Human IgG Fc-HRP (Jackson ImmunoResearch, applicable for ELISA)
https://www.jacksonimmuno.com/catalog/products/109-035-098

Goat Anti-Mouse IgG Fc-HRP (Jackson ImmunoResearch, applicable for ELISA)
https://www.jacksonimmuno.com/catalog/products/115-035-071

Eukaryotic cell lines

Policy information about cell lines

Cell line source(s)	CHO-K1 cells, HEK293T cells, Vero E6 cells, Raji cells, THP-1 cells and K562 cells were from ATCC. Huh7 cells were from Institute of Basic Medical Sciences, CAMS.
Authentication	No cell lines were authenticated. All cells were purchased commercially and are not misidentified.
Mycoplasma contamination	All cell lines have been tested negative for mycoplasma contamination.
Commonly misidentified lines (See ICLAC register)	No commonly misidentified cell lines were used.

Animals and other organisms

Policy information about studies involving animals; ARRIVE guidelines recommended for reporting animal research

Laboratory animals	Nine 6-7 years old rhesus monkeys (3 females and 6 males) were used in this study.
Wild animals	This study did not involve wild animals.
Field-collected samples	This study did not involve samples collected from the field.
Ethics oversight	Monkey studies were carried out in an animal biosafety level 4 (ABSL-4) facility with protocols approved by the Laboratory Animal Welfare and Ethics Committee of the Chinese Academy of Sciences.

Note that full information on the approval of the study protocol must also be provided in the manuscript.

Human research participants

Policy information about studies involving human research participants

Population characteristics	We used the blood from one convalescent COVID-19 patient (Male, 40 years old) in China.
Recruitment	The patient agreed to provide the biospecimen for detection, further diagnostic and scientific research when hospitalization.
Ethics oversight	Shantou University

Note that full information on the approval of the study protocol must also be provided in the manuscript.

Flow Cytometry

Plots

Confirm that:

- [x] The axis labels state the marker and fluorochrome used (e.g. CD4-FITC).
- [x] The axis scales are clearly visible. Include numbers along axes only for bottom left plot of group (a 'group' is an analysis of identical markers).
- [x] All plots are contour plots with outliers or pseudocolor plots.
- [x] A numerical value for number of cells or percentage (with statistics) is provided.

Methodology

Sample preparation	Raji, THP-1 and K562 cells were collected and washed three times with cold PBS buffer.
Instrument	CytoFLEX (Beckman Coulter)
Software	The software CytExpert was used for data collection and analysis.

Cell population abundance	For Raji, THP-1 and K562 cells, more than 95% of the cells are live cells.
Gating strategy	Dead cells were excluded using FSC/SSC gates. A human IgG control was inclueded in this study. The positive boundary was defined, by wihch, less than 3 % of cells in the control group are positive.

[x] Tick this box to confirm that a figure exemplifying the gating strategy is provided in the Supplementary Information.
